# Cannabis use among Canadian veterans: associations with the use of other substances, chronic pain conditions, mental disorders, suicide behaviours, and help-seeking

**DOI:** 10.1186/s42238-025-00377-6

**Published:** 2026-01-19

**Authors:** Tamara L. Taillieu, Samantha Salmon, Ashley Stewart-Tufescu, Jitender Sareen, Murray W. Enns, Natalie Mota, Shay-Lee Bolton, R. Nicholas Carleton, Murray B. Stein, Tracie O. Afifi

**Affiliations:** 1 Department of Community Health Sciences, University of Manitoba, 438-727 McDermot Avenue, Winnipeg, MB R3E 3P5 Canada; 2 Department of Community Health Sciences, University of Manitoba, S112-750 Bannatyne Avenue, Winnipeg, MB R3E 0W2 Canada; 3 Faculty of Social Work, University of Manitoba, 418 A Tier Building, 173 Dafoe Road, Winnipeg, MB R3T 2N2 Canada; 4 Departments of Psychiatry, Psychology, and Community Health Sciences, University of Manitoba, PZ430-771 Bannatyne Avenue, Winnipeg, MB R3E 3N4 Canada; 5 Department of Psychiatry, University of Manitoba, PZ430-771 Bannatyne Avenue, Winnipeg, MB R3E 3N4 Canada; 6 Departments of Clinical Health Psychology and Psychiatry, University of Manitoba, PZ350-771 Bannatyne Avenue, Winnipeg, MB R3E 3N4 Canada; 7 Departments of Psychiatry and Community Health Sciences, University of Manitoba, PZ430-771 Bannatyne Avenue, Winnipeg, MB R3E 3N4 Canada; 8 Anxiety and Illness Behaviours Laboratory, Department of Psychology, University of Regina, Regina, SK S4S 0A2 Canada; 9 Department of Psychiatry and School of Public Health, University of California San Diego, 9500 Gilan Drive, La Jolla, San Diego, CA 92093 USA; 10 Department of Community Health Sciences and Department of Psychiatry, University of Manitoba, S113-750 Bannatyne Avenue, Winnipeg, MB R3E 0W2 Canada

**Keywords:** Cannabis, Marijuana, Veterans, Mental disorders, Substance use, Perceived need for care, Professional help-seeking

## Abstract

**Background:**

A substantial proportion of Veterans experience mental and physical health difficulties, including post-traumatic stress disorder and chronic pain, for which cannabis is sometimes medically authorized. However, relatively little is known about cannabis use among Canadian Veterans. Information on the prevalence of use, as well as the mental health profiles and help-seeking behaviours of Veterans who use cannabis, is important for developing more targeted prevention and intervention services and supports.

**Methods:**

The current study used data from Veteran respondents (*n* = 1992) of the 2018 Canadian Armed Forces Members and Veterans Mental Health Follow-up Survey. Descriptive statistics and logistic regression models were used to examine associations between past 12-month cannabis use and concurrent mental disorders, chronic pain conditions, use of other substances, suicide behaviours, perceived need for care, and help-seeking behaviours.

**Results:**

Several participating Veterans (16.7%) reported using cannabis in the past 12-months. Infrequent use of cannabis in the past 12 months was associated with statistically significantly increased odds of current smoking, binge drinking, and post-traumatic stress disorder in fully adjusted models (Adjusted odds ratios [AOR-2] = 1.93, 2.58, and 2.37, respectively). Regular use of cannabis in the past 12 months was associated with statistically significantly increased odds of current tobacco smoking, arthritis and any chronic pain condition, several mental health disorders, and suicide ideation in fully adjusted models (AOR-2 ranged from 1.61 to 3.99). Veterans who used cannabis both infrequently and regularly were also statistically significantly more likely to perceive a need for care (AOR-2 = 2.15 and 4.85, respectively) and report professional help-seeking (AOR-2 = 2.25 and 5.56, respectively) in the past 12-months compared to Veterans who did not use cannabis.

**Conclusions:**

The strong association of past 12-month cannabis use with nicotine, chronic pain, mental disorders, and suicide ideation suggests that Veterans who use cannabis regularly may represent a population with complex needs who may benefit from additional services and supports. Cannabis using Veterans were also more likely to perceive a need for and seek professional help, which provides opportunities for more timely identification and intervention.

The mental health and well-being of military personnel and Veterans is an important public health priority. Research has indicated that a substantial proportion of Canadian Veterans experience mental and/or physical health difficulties (Mahar et al. 2018; St. Cyr et al. 2022; Thompson et al. 2016; VanTil et al. 2018). Almost one-quarter of Canadian Armed Forces (CAF) Veterans self-report having been diagnosed with a mental disorder, including mood disorders (17.1%), anxiety disorders (11.1%), and post-traumatic stress disorder (PTSD; 13.1%) (Thompson et al. 2016). CAF Veterans also report a higher prevalence of physical health conditions, particularly conditions associated with chronic pain, than their civilian counterparts (Thompson et al. 2016; VanTil et al. 2018; Perera et al. 2021). PTSD and chronic pain are conditions for which cannabis is sometimes medically authorized (Reyes-Vélez et al. 2022; Turner and MacKillop 2021).

In Canada, the medical use of cannabis was legalized in 2001, with medical access dramatically increasing over time due to legislative changes in 2014 and 2017 (Turner and MacKillop 2021). By 2016, more than 1,700 Canadian Veterans were being reimbursed for medical cannabis, at an annual cost of about $20 million (Reyes-Vélez et al. 2022). Non-medical use of cannabis has also been increasing over time in Canada (Lowry and Corsi 2020) and elsewhere (Hasin 2018). However, most research on cannabis use among Veterans is from the United States (US) (Turner and MacKillop 2021). Differences in the accessibility of cannabis to treat mental health and chronic pain conditions, health care systems and access, and/or social norms around cannabis use suggest that research specific to the Canadian context is warranted (Goundar et al. 2021; Lancione et al. 2020).

Research has indicated that cannabis use is highly comorbid with mental health disorders and other substance use among Veterans (Turner and MacKillop 2021; Davis et al. 2018; Ecker et al. 2020; Hill et al. 2021; Sterniczuk and Whelan 2016). For example, in a nationally representative sample of US Veterans, lifetime cannabis use was associated with lifetime PTSD, as well as lifetime depressive, anxiety, and substance use disorders (Hill et al. 2021). Another large study of US Veterans diagnosed with cannabis use disorder (CUD) found most were also diagnosed with a mental disorder (79.1%) or another substance use disorder (76.8%) (Ecker et al. 2020). Nicotine dependence, alcohol use disorder, and other drug use also increases the odds of past-year cannabis use among US Veterans (Davis et al. 2018). Meta-analytic results also indicate an association between heavy cannabis use and suicidal behaviors (Borges et al. 2016). Less research exists examining cannabis use among Canadian Veterans. In fact, a recent review examining the correlates and consequences of cannabis use among Veterans found that 93.0% of the studies were conducted in the US (Turner and MacKillop 2021), and research on US Veterans may not be generalizable to Canadian Veterans. More research on the mental health profiles of Canadian Veterans who use cannabis might also be useful for identifying those who may benefit from additional supports and services.

Among US Veterans, comorbid CUD and mental health disorder was associated with greater mental health care utilization (Ecker et al. 2020). US Veterans with lifetime cannabis use or CUD were more likely to seek mental health treatment than those who never used cannabis (Hill et al. 2021). In a small sample of CAF Veterans receiving treatment for PTSD, 51.7% of the sample reported a history of cannabis use, and among cannabis users, 42% reported initiating use after release from the CAF (Sterniczuk and Whelan 2016). In another sample of Canadian Veterans with chronic pain, 63.9% of the sample reported current cannabis use (Sheehy et al. 2025). These findings suggest that a substantial proportion of Canadian Veterans seeking help for PTSD or chronic pain might also be using cannabis. CAF military personnel and Veterans report a greater perceived need for care and a higher prevalence of help-seeking than the Canadian general population (Sareen et al. 2016; Fikretoglu et al. 2016; MacLean et al. 2021). However, potential differences in perceived need for care and professional help-seeking behaviours among Canadian Veterans who use and who do not use cannabis remains unknown.

Child abuse history, exposure to deployment-related trauma events (DRTEs), and chronic pain conditions have been associated with mental disorders (Afifi et al. 2021a; Higgins et al. 2014; Taillieu et al. 2022; Rebeira et al. 2017) and cannabis use (Afifi et al. 2023; Mannes et al. 2023) among military personnel and Veterans. In a sample of Canadian Veterans, experiencing physical abuse, sexual abuse, and/or neglect in childhood was associated with increased odds of past 12-month cannabis use (Afifi et al. 2021a). The study also reported that exposure to DRTEs also increased the odds of past 12-month cannabis use (Afifi et al. 2021a). However, in a small sample of Veterans in treatment for PTSD, current cannabis use was not significantly associated with the number of tours served, whether they had sustained an injury during service, or whether they were medically released or not (Sterniczuk and Whelan 2016). In this sample, many Veterans (21%) reported using cannabis to manage and reduce pain, although pain relief motivations for use were not significantly related to pain severity scores (Sterniczuk and Whelan 2016). Similar findings have been reported in a sample of CAF Veterans living with chronic pain; that is, many (45.7%) report using cannabis for pain management (i.e., to reduce opioid use) (Harris-Lane et al. 2025). There are also sex differences associated with child abuse history, DRTEs, and mental disorders (Taillieu et al. 2022; Rebeira et al. 2017; Afifi et al. 2014), all of which impact help-seeking behaviours (St. Cyr et al. 2022; MacLean et al. 2021; Turner et al. 2017). However, whether these factors (i.e., child maltreatment history, exposure to DRTEs, chronic pain, and sex) moderate the relationship between cannabis use and perceived need for care and help-seeking behaviours in Veterans remains unknown. We hypothesize that potential differences in the strength of the association between cannabis use and perceived need for care and help-seeking exist, with stronger associations noted for those with traumatic exposures (i.e., child maltreatment or deployment trauma exposures), chronic pain conditions, and mental health problems.

Therefore, the current study was designed to examine: (1) the association between past 12-month cannabis use (infrequent use and regular use) and concurrent use of other substances (tobacco smoking, alcohol use, binge drinking), chronic pain conditions (arthritis, back problems, migraine headaches, gastrointestinal conditions), mental health disorders (PTSD, major depressive episode, generalized anxiety disorder, social phobia, panic disorder), and suicide behaviours (ideation, plans, attempts); (2) whether past 12-month cannabis use (infrequent use and regular use) was associated with an increased likelihood of past 12-month perceived need for care and professional help-seeking behaviours; and (3) whether respondent sex, child maltreatment history, any DRTE, any chronic pain condition, or any past 12-month mental disorder moderated the association between past 12-month cannabis use (infrequent use and regular use) and perceived need and help-seeking in a sample of Canadian Veterans.

## Methods

### Sample

Data were from the Canadian Armed Forces Members and Veterans Mental Health Follow-Up Survey (CAFVMHS) collected by Statistics Canada between January and May 2018 (Afifi et al. 2021b). The CAFVMHS is a follow-up survey of CAF Regular Force members originally interviewed in 2002 as part of the Canadian Community Health Survey – Canadian Forces Supplement, which included a representative sample of 5,155 active duty Regular Forces CAF personnel. The CAFVMHS target population included Regular Forces personnel originally interviewed in 2002. In 2018, Statistics Canada re-interviewed 68.7% (*n* = 2,941) of those eligible (i.e., not deceased, still living in one of the 10 Canadian provinces, not recently interviewed for other Statistics Canada surveys, and not part of a random subset of respondents excluded because of budgetary constraints) (Bolton et al. 2021). Therefore, the CAFVMHS included those still actively serving Regular Forces personnel at the time of the interview in 2018 (*n* = 949) as well as those who had left the CAF (i.e., Veterans) since 2002 (*n* = 1,992; 66% of the original sample were Veterans at the follow-up in 2018). The current study only included Veterans since questions on cannabis use were only asked among Veterans in the CAFVMHS. More details on the CAFVMHS methods have been published elsewhere (Afifi et al. 2021b; Bolton et al. 2021).

### Measures

#### Cannabis use

Cannabis use was assessed with a question asking respondents if they had ever tried marijuana or hashish (lifetime use), and if they had used marijuana or hashish in the past 12-months. Respondents were coded into one of three categories based on the frequency of cannabis use in the past 12-months: (1) No use (coded as 0); (2) Infrequent use (less than once a month, 1 to 3 times a month, or once a week; coded as 1); and (3) Regular use (more than once a week, every day; coded as 2). Respondents indicating that they had only used marijuana or hashish once were coded into the “no” category (as one-time users are likely different from those using cannabis more often) (Afifi et al. 2023).

#### Other substance use

Other substance use included current nicotine smoking, any past 12-month alcohol use, and any past 12-month binge drinking. Current nicotine smoking was assessed with the question: “At the present time, do you smoke cigarettes daily, occasionally, or not at all?” Respondents were coded as “yes” (coded as 1) to current smoking if they reported daily or occasional smoking (no current smoking coded as 0). Past 12-month alcohol use was assessed with the question: “During the past 12 months, have you had a drink of beer, wine, liquor, or any other alcoholic beverage” (no = 0 or yes = 1). Past 12-month binge drinking was assessed with the question: “How often in the past 12 months have you had 5 or more drinks on one occasion?” (never, less than once a month, once a month, 2 to 3 times a month, once a week, more than once a week). Respondents were coded as “yes” (coded a 1) to past 12-month binge drinking if they reported drinking 5 or more drinks on any occasion of ‘less than once a month’ or more (i.e., any response other than ‘never’ to the question; “no” responses were coded a 0).

#### Chronic pain conditions

Chronic pain conditions included arthritis, back problems, migraine headaches, and/or gastrointestinal conditions (i.e., irritable bowel syndrome, inflammatory bowel disease, intestinal or stomach ulcers). Respondents were coded as “yes” (coded as 1) if they self-reported being diagnosed by a health professional with any of the aforementioned long-term conditions (which had lasted or were expected to last 6 months or more; “no” responses were coded as 0). An ‘any chronic pain condition’ (no = 0 or yes = 1) variable was also computed based on whether the respondent reported seeking care for any of the chronic pain conditions assessed in the survey.

#### Mental disorders

Past 12-month mental disorders included PTSD, major depressive disorder, generalized anxiety disorder, social phobia, and panic disorder and were assessed using the World Health Organization version of the Composite International Diagnostic Interview (CIDI) based on DSM-IV diagnostic criteria (no = 0 or yes = 1) (Kessler et al. 2004; Kessler and Üstün 1994; World Health Organization 1994;). Past 12-month PTSD was not directly assessed; therefore, an algorithm was developed based on several variables to assess past 12-month PTSD symptoms and has been used for other studies (Afifi et al. 2021a; Sareen et al. 2021; Easterbrook et al. 2023). The algorithm required respondents to have met CIDI-based criteria for PTSD between 2002 and 2018 (i.e., since last interview diagnosis), respond “yes” to a question asking about PTSD-related reactions in the past 12 months, and report experiencing at least 3 of the 7 PTSD symptoms that were assessed in relation to the past 12 month timeframe (Sareen et al. 2021). An ‘any mental disorder’ variable was also computed based on whether the respondent met diagnostic criteria for any of the mental disorders assessed in the past 12-months (no = 0 or yes = 1).

#### Suicide behaviours

Suicide ideation was assessed with a question asking whether the respondent had seriously thought about attempting suicide or taking their own life in the past 12-months (no = 0 or yes = 1). Suicide plans were assessed with a question asking whether the respondent had made a plan to attempt suicide in the past 12-months (no = 0 or yes = 1). The low prevalence of past 12-month suicide attempts precluded the examination of this type of suicide behaviour in the current study.

#### Perceived need for care

Perceived need for care was assessed with a modified version of the Perceived Need for Care Questionnaire (PNCQ) (Meadows et al. 2000). The PNCQ categorizes respondents into 1 of 4 levels of perceived need (i.e., no need, needs fully met, needs partially met, needs not met) for help with their emotions, mental health, or use of alcohol or drugs across different domains of need (i.e., information, medication, counselling, financial or housing assistance, employment assistance, personal relationship) in the past 12-months (0 = no need or 1 = perceived a need for care). Dichotomous coding was used to classify respondents based on whether they perceived a need for care (no need = 0 vs. need = 1) regardless of whether the need was met, partially met, or not met in each of the individual domains as well as overall across all domains (i.e., any perceived need in any domain).

#### Professional help-seeking

A series of questions assessed whether respondents had sought help from different sources for a problem with their emotions, mental health, or use of alcohol or drugs in the past 12-months. Professional help-seeking included help from a psychiatrist; psychologist; family doctor, general practitioner, or medical officer; other medical doctor; nurse, nurse practitioner, physician assistant, or medic; and/or social worker, counsellor, or psychotherapist (no = 0 or yes = 1). An any professional help-seeking variable was also computed based on whether the respondent reported seeking any professional help in the past 12-months (no help-seeking = 0 or any professional help-seeking = 1).

#### Child maltreatment

Child maltreatment history included experiencing physical abuse, sexual abuse, emotional abuse, neglect, and/or exposure to intimate partner violence in the home before the age of 16 years based on thresholds described elsewhere (Afifi et al. 2021a; Afifi et al. 2023). A dichotomous ‘any child maltreatment’ (no child maltreatment = 0 or any child maltreatment = 1) variable was computed based on whether the respondent reported having experienced one or more of the types of child maltreatment assessed in the current study.

#### DRTEs

Exposure to DRTEs during a CAF deployment was assessed with a series of items from a modified version of the Deployment Experiences Scale (Afifi et al. 2021b). DRTEs were assessed with 10 dichotomous items (no = 0 or yes = 1), including having ever been sexually assaulted while on a CAF deployment; unwanted sexual touching while on a CAF deployment; known someone seriously injured or killed; been in a life threatening situation where you were unable to respond due to rules of engagement; seen ill or injured women or children who you were unable to help; received incoming artillery, rocket, or mortar fire; felt responsible for the death of Canadian or ally personnel; ever had a close call, for example shot or hit but protective gear saved you. A dichotomous any DTRE variable (no DRTEs = 0 or any DRTE = 1) was computed based on whether the respondent reported having experienced one or more of the DRTEs assessed in the current study.

#### Sociodemographic Covariates

Sociodemographic and military covariates included sex (male = 1 or female = 2), age (continuous), race/ethnicity (white = 0 or not white = 1), marital status (married or common-law = 1; separated, divorced, or widowed = 2; single, never married = 3), total household income in past year (1 = less than $50,000; 2 = $50,000 to $99,999; 3 = $100,000 to $149,999; or 4 = $150,000 or more), highest level of education (1 = less than high school, 2 = high school diploma or equivalent, 3 = some post-secondary [less than a Bachelor’s degree], 4 = Bachelors’ degree or higher), and last military environment (1 = Air, 2 = Land, or 3 = Sea).

#### Statistical analyses

Descriptive statistics were used to describe sample characteristics, and to examine the prevalence of substance use, chronic pain conditions, mental health disorders, suicide behaviours, perceived need for care, and help-seeking behaviours by past 12-month cannabis use. Logistic regression models were computed to examine the association of past 12-month cannabis use (independent variable) with substance use, chronic pain conditions, mental health disorders, and suicide behaviours (dependent variables). No past 12-month cannabis use was the reference category (relative to infrequent cannabis use and regular cannabis use). All models were first run adjusting for sociodemographic covariates, and then further adjusted for any child maltreatment and any DRTE. Interaction terms were entered into the perceived need for care and help-seeking models (moderator x cannabis use) to examine whether sex, any child maltreatment history, any DRTE, any past 12-month mental disorder, or any chronic pain condition moderated the association between cannabis use and perceived need for care or help-seeking behaviour.

Data were weighted to ensure that estimates were representative of the original 2002 CAF study sample (as a representative sample of full-time Regular Forces personnel was part of the original sampling frame in 2002 and formed the basis for the sampling frame for the follow-up survey in 2018). The longitudinal weights computed by Statistics Canada included adjustments to the initial sample weights to reflect those included in 2018 (e.g., suppression of out-of-scope units and adjustments for non-response). Bootstrapping was used as the variance estimation technique to account for the complex survey design. Further details on the CAFVMHS methodology have been published elsewhere (Afifi et al. 2021b; Bolton et al. 2021). Results at *p* < 0.05 were considered statistically significant.

## Results

Past 12-month cannabis use was 16.7%. Among participants reporting past 12-month cannabis use, 31.5% reported using cannabis less than once a month, 10.3% reported using cannabis 1 to 3 times a month, 3.0% reported using cannabis once a week, 11.7% reported using cannabis more than once a week, and 43.6% reported using cannabis every day. The sample sociodemographic characteristics are provided in Table 1.

**Table 1:** Sample sociodemographic characteristics

Covariate	% (95% CI)
Sex	
Male	88.1 (87.4, 88.7)
Female	11.9 (11.3, 12.6)
Age (mean, SE)	53.6 (0.16)
Race/Ethnicity	
White	95.0 (93.6, 96.0)
Non-white	5.0 (4.0, 6.4)
Marital Status 2018	
Married/common-law	81.8 (79.8, 83.6)
Single, never married	6.5 (5.4, 7.9)
Separated, divorced, widowed	11.7 (10.2, 13.4)
Household Income 2018	
Less than $50,000	6.8 (5.6, 8.2)
$50,000 to $99,999	35.3 (33.0, 37.7)
$100,000 to $149,999	27.7 (25.6, 29.9)
$150,000 or more	30.2 (28.1, 32.4)
Highest Level of Education 2018	
Less than high school	4.7 (3.8, 5.8)
High school diploma or equivalent	37.6 (35.4, 39.9)
Some post-secondary (less than a Bachelor’s degree)	40.1 (37.7, 42.5)
Bachelor’s degree or higher	17.6 (16.4, 18.9)
Last Military Environment	
Air	35.1 (32.9, 37.3)
Land	46.2 (43.8, 48.6)
Sea	18.8 (16.9, 20.8)
Any child maltreatment	
No	38.1 (35.8, 40.5)
Yes	61.9 (59.5, 64.2)
Any deployment-related trauma	
No	33.9 (31.7, 36.2)
Yes	66.1 (63.8, 68.3)
Any mental health disorder	
No	83.3 (81.2, 85.2)
Yes	16.7 (14.8, 18.8)
Any chronic pain condition	
No	32.8 (30.6, 35.0)
Yes	67.2 (65.0, 69.4)

Notes. *CI* Confidence interval, *SE* Standard error

Association between past 12-month cannabis use and the use of other substances, chronic pain conditions, mental health disorders, and suicide-related behaviours are provided in Table 2. Infrequent past 12-month cannabis use was statistically significantly associated with being a current smoker (adjusted odds ratio [AOR]−1 = 1.90, 95% confidence interval [CI] = 1.07–3.37) and past 12-month binge drinking (AOR-1 = 2.66, 95% CI = 1.62–4.36) in models adjusting for socioeconomic covariates. AORs were attenuated, but remained statistically significant for infrequent cannabis use and current smoking and past 12-month binge drinking after further adjustment for any child maltreatment and any DRTE (AOR-2 = 1.93, 95% CI = 1.08–3.47 and 2.58, 95% CI = 1.57–4.24, respectively). Regular past 12-month cannabis use was statistically significantly associated with being a current smoker after adjustment for sociodemographic covariates (AOR-1 = 1.75, 95% CI = 1.07, 2.86) and after further adjustment for any child maltreatment and any DRTE (AOR-2 = 1.78, 95% CI = 1.09, 2.91).

**Table 2:** Associations between Past 12-Month Cannabis Use and Concurrent Substance Use, Chronic Pain Conditions, Mental Disorders, and Suicide-Related Behaviours among Canadian Veterans

	No Use	Infrequent Use	Regular Use	AOR-1^a ^(95% CI)		AOR-2^a^ (95% CI)	
	% (95% CI)	% (95% CI)	% (95% CI)	Infrequent Use	Regular Use	Infrequent Use	Regular Use
Substance Use							
Current smoker	13.2 (11.3, 15.3)	25.2 (16.8, 35.9)	28.9 (21.5, 37.7)	1.90* (1.07, 3.37)	1.75* (1.07, 2.86)	1.93* (1.08, 3.47)	1.78* (1.09, 2.91)
Any alcohol use^b^	89.5 (87.7, 91.1)	92.8 (89.2, 95.3)	_ _ _	1.59 (0.93, 2.72)	_ _ _	1.64 (0.95, 2.83)	___
Any binge drinking	52.5 (49.8, 55.3)	74.5 (65.6, 81.8)	60.9 (51.8, 69.4)	2.66*** (1.62, 4.36)	1.22 (0.80, 1.87)	2.58*** (1.57, 4.24)	1.19 (0.77, 1.83)
Chronic Pain Conditions							
Arthritis	37.9 (35.5, 40.3)	38.2 (29.4, 47.8)	52.0 (43.0, 60.9)	1.11 (0.72, 1.71)	1.75** (1.17, 2.63)	1.12 (0.73, 1.74)	1.68* (1.10, 2.56)
Back problems	48.1 (45.5, 50.8)	53.8 (44.0, 63.3)	60.7 (51.4, 69.3)	1.51 (0.98, 2.33)	1.40 (0.92, 2.14)	1.41 (0.92, 2.18)	1.28 (0.83, 1.97)
Migraine headaches	11.3 (9.7, 13.1)	15.8 (10.0, 24.1)	19.9 (14.0, 27.4)	1.45 (0.74, 2.81)	1.84* (1.07, 3.19)	1.43 (0.73, 2.80)	1.69 (0.96, 2.99)
Gastrointestinal condition	11.9 (10.3, 13.7)	9.3 (4.9, 16.9)	22.3 (15.8, 30.6)	0.78 (0.38, 1.59)	1.82* (1.10, 3.00)	0.78 (0.38, 1.60)	1.68 (0.99, 2.84)
Any chronic pain condition	66.0 (63.5, 68.4)	66.5 (57.1, 74.7)	79.2 (71.1, 85.5)	1.23 (0.79, 1.90)	1.77* (1.11, 2.81)	1.20 (0.77, 1.86)	1.61* (1.01,2.56)
Mental Disorders							
Major depressive episode	18.1 (16.1, 20.3)	21.0 (13.9, 30.4)	48.8 (40.3, 57.3)	1.29 (0.70, 2.38)	3.23*** (2.11, 4.94)	1.18 (0.64, 2.17)	3.09*** (2.02, 4.71)
Generalized anxiety disorder	6.2 (5.0, 7.6)	12.2 (7.0, 20.5)	27.0 (19.8, 35.7)	2.12 (0.99, 4.55)	3.91*** (2.16, 7.10)	1.89 (0.86, 4.16)	3.56*** (1.96, 6.47)
Social phobia	12.5 (10.7, 14.4)	19.4 (12.3, 29.2)	37.5 (29.6, 46.0)	1.76 (0.96, 3.21)	3.24*** (2.04, 5.13)	1.73 (0.94, 3.18)	3.11*** (1.94, 4.97)
Panic disorder	10.5 (8.9, 12.4)	13.0 (7.3, 22.0)	28.0 (20.4, 37.2)	1.39 (0.67, 2.91)	2.01** (1.19, 3.39)	1.25 (0.59, 2.65)	1.82* (1.07, 3.08)
Post-traumatic stress disorder	9.2 (7.7, 11.0)	17.9 (11.3, 27.1)	31.3 (23.2, 40.8)	2.60** (1.30, 5.18)	3.12*** (1.81, 5.39)	2.37* (1.16, 4.84)	2.92*** (1.70, 5.02)
Any mental disorder	27.0 (24.7, 29.5)	34.1 (25.1, 44.4)	66.6 (57.8, 74.3)	1.46 (0.88, 2.42)	4.17*** (2.62, 6.65)	1.31 (0.79, 2.19)	3.99*** (2.50, 6.38)
Suicide-Related Behaviours							
Suicide ideation	8.3 (6.8, 9.9)	14.3 (8.6, 22.6)	25.8 (18.9, 34.1)	2.21* (1.09, 4.51)	2.91*** (1.65, 5.15)	1.92 (0.95, 3.89)	2.68*** (1.50, 4.79)
Suicide plan^a^	3.9 (2.9, 5.2)	7.1 (4.4, 11.2)	_ _ _	1.76 (0.88, 3.52)	_ _ _	1.62 (0.82, 3.21)	___
Suicide attempt	NR	NR	NR	NR	NR	NR	NR

Notes. CI = confidence interval; AOR = adjusted odds ratio. AOR-1 models adjust for sex, race, marital status, total household income, education, and last military environment. AOR-2 models adjust for AOR-1 plus any child maltreatment and any deployment-related traumatic event. NR = not releasable (i.e., analyses regarding past 12-month suicide attempts were not released as per Statistics Canada data release guidelines to protect participant confidentiality)

^a^No cannabis use in the past 12-months was the reference category with an odds of 1.00

^b^To meet Statistics Canada data release guidelines, we were only able to examine past 12-month alcohol use and suicide plans using a dichotomous version of the past 12-month cannabis use variable (i.e., no use/1 time use vs. used more than 1 time)

^*^p$$\le$$.05; **p$$\le$$.01; ***p$$\le$$.001

Infrequent past 12-month cannabis use was not significantly associated with any of the chronic pain conditions assessed in this study. Regular cannabis use in the past 12 months was associated with arthritis, migraine headaches, gastrointestinal conditions, and any chronic pain condition in models adjusting for sociodemographic covariates (AOR-1 ranged from 1.75 to 1.84). Associations between regular use of cannabis in the past 12 months and arthritis (AOR-2 = 1.68, 95% CI = 1.10–2.56) and any chronic pain condition (AOR-2 = 1.61, 95% CI = 1.01–2.56) remained statistically significant in models further adjusting for any child maltreatment and any DRTE.

Infrequent past 12-month cannabis use was statistically significantly associated with past 12-month PTSD in models adjusting for sociodemographic covariates (AOR-1 = 2.60, 95% CI = 1.30, 5.18) and after further adjustments for any child maltreatment and any DRTE (AOR-2 = 2.37, 95% CI = 1.16, 4.84). Regular use of cannabis in the past 12 months was significantly associated with past 12-month major depressive episodes, generalized anxiety disorder, social phobia, panic disorder, and PTSD in models adjusting for sociodemographic covariates (AOR-1 ranged from 2.01 to 4.17). AORs were attenuated, but remained statistically significant, after further adjustment for any child maltreatment and any DRTE (AOR-2 ranged from 1.82 to 3.99).

Infrequent past 12-month cannabis use was significantly associated with past 12-month suicide ideation (AOR-2.21, 95% CI = 1.09, 4.51) in models adjusting for sociodemographic covariates, but not in fully adjusted models. Regular use of cannabis in the past 12-months was significantly associated with past 12-month suicide ideation (AOR-1 = 2.91, 95% CI = 1.65–5.16) in models adjusting for sociodemographic covariates, and after further adjustment for any child maltreatment and any DRTE (AOR-2 = 2.68, 95% CI = 1.50–4.79).

Associations between past 12-month cannabis use and perceived need for care are provided in Table 3. Infrequent past 12-month cannabis use was associated with increased odds of perceiving a need for information, medication, counselling, and having any perceived need for care in models adjusting for sociodemographic covariates (significant AOR-1 ranged from 1.69 to 2.44). Associations between infrequent cannabis use and perceiving a need for information, medication, and any perceived need remained statistically significant after further adjustment for any child maltreatment and any DRTE (significant AOR-2 ranged from 1.72 to 2.21). With the exception of a perceived need for employment assistance, regular use of cannabis in the past 12-months was statistically significantly associated with all of the other domains of need assessed in models adjusting for sociodemographic covariates (significant AOR-1 ranged from 2.14 to 7.63) and after further adjustment for any child maltreatment and any DRTE (significant AOR-2 ranged from 1.95 to 7.30).

**Table 3:** Associations between Past 12-Month Cannabis Use and Perceived Need for Care in Past 12-Months

Perceived Need (PN) for Care Domain	No Use	Infrequent Use	Regular Use	AOR-1^a^ (95% CI)		AOR-2^a^ (95% CI)	
	% (95% CI)	% (95% CI)	% (95% CI)	Infrequent Use	Regular Use	Infrequent Use	Regular Use
PN for information	19.0 (16.9, 21.3)	32.6 (24.1, 42.5)	47.7 (39.4, 56.2)	1.83* (1.10, 3.04)	2.90*** (1.88, 4.48)	1.72* (1.01, 2.92)	2.64*** (1.69, 4.12)
PN for medication	18.4 (16.4, 20.6)	33.5 (24.6, 43.7)	66.5 (57.4, 74.5)	2.44*** (1.46, 4.07)	7.63*** (4.69, 12.42)	2.21** (1.31, 3.75)	7.30*** (4.41, 12.10)
PN for counselling	24.6 (22.3, 27.0)	36.7 (27.6, 46.9)	63.5 (54.9, 71.3)	1.69* (1.03, 2.78)	4.30*** (2.79, 6.63)	1.58 (0.96, 2.60)	4.00*** (2.58, 6.20)
PN for financial or housing assistance	5.6 (4.4, 7.1)	11.7 (6.5, 20.2)	18.4 (12.3, 26.4)	2.34 (0.95, 5.77)	2.32* (1.21, 4.45)	2.14 (0.84, 5.46)	2.18* (1.14, 4.17)
PN for employment assistance	7.7 (6.3, 9.4)	9.2 (4.9, 16.5)	13.8 (8.7, 21.1)	1.21 (0.50, 2.90)	1.31 (0.70, 2.46)	1.11 (0.47, 2.64)	1.25 (0.64, 2.42)
PN for help with personal relationships	10.4 (8.9, 12.2)	10.6 (6.0, 18.0)	24.3 (17.6, 32.4)	1.12 (0.58, 2.18)	2.14** (1.28, 3.56)	1.00 (0.52, 1.94)	1.95* (1.15, 3.31)
Any PN (across all domains)	37.0 (34.4, 39.6)	58.1 (48.6, 66.9)	79.6 (71.8, 85.6)	2.32*** (1.49, 3.60)	5.11*** (3.15, 8.29)	2.15*** (1.35, 3.42)	4.85** (2.97, 7.91)

Notes.  CI = confidence interval; AOR = adjusted odds ratio. AOR-1 models adjust for sex, race, marital status, total household income, education, and last military environment. AOR-2 models adjust for AOR-1 plus any child maltreatment and deployment-related traumatic event

^a^No cannabis use in the past 12-months was the reference category with an odds of 1.00

^*^p$$\le$$.05; **p$$\le$$.01; ***p$$\le$$.001

Associations between past 12-month cannabis use and past 12-month professional help seeking behaviours are provided in Table 4. In models adjusting for sociodemographic covariates, infrequent and/or any past 12-month cannabis use was statistically significantly associated with seeking help from a family doctor, general practitioner, or medical officer; other medical doctor (e.g., cardiologist, gynecologist, urologist); a nurse, nurse practitioner, physician assistant, or medic: and any professional help-seeking behaviour in the past 12-months (AOR-1 ranged from 2.38 to 3.17), and remained statistically significant after further adjustment for any child maltreatment and any DRTE (AOR-2 ranged from 2.25 to 3.49). Regular use of cannabis in the past 12-months was also associated with any past 12-month help-seeking (AOR-1 = 5.76, 95% CI = 3.59–9.23) and across a range of providers (AOR-1 ranged from 2.47 to 3.88) in models adjusting for sociodemographic covariates. Associations were slightly attenuated, but remained statistically significant, after further adjustment for any child maltreatment and any DRTE (AOR-2 ranged from 2.30 to 5.56).

**Table 4:** Associations between Past 12-Month Cannabis Use and Professional Help-Seeking in Past 12-Months

Type of Professional	No Use	Infrequent Use	Regular Use	AOR-1^a^ (95% CI)		AOR-2^a^ (95% CI)	
	% (95% CI)	% (95% CI)	% (95% CI)	Infrequent Use	Regular Use	Infrequent Use	Regular Use
Psychiatrist	7.8 (6.3, 9.5)	8.4 (4.3, 16.0)	23.9 (17.4, 31.9)	1.25 (0.53, 2.93)	2.47*** (1.42, 4.30)	1.15 (0.49, 2.73)	2.30** (1.29, 4.12)
Psychologist	13.6 (11.7, 15.6)	17.7 (11.2, 26.8)	37.3 (29.5, 45.9)	1.63 (0.84, 3.17)	2.95*** (1.83, 4.76)	1.55 (0.78, 3.06)	2.76*** (1.68, 4.54)
Family doctor, general practitioner, or medical officer	15.0 (13.2, 17.1)	27.0 (19.0, 36.9)	43.6 (35.0, 52.6)	2.57*** (1.54, 4.29)	3.88*** (2.42, 6.20)	2.42*** (1.45, 4.03)	3.62*** (2.25, 5.83)
Other medical doctor (e.g., cardiologist, gynecologist, urologist)^b^	2.2 (1.5, 3.2)	6.9 (4.3, 10.9)	_ _ _	3.17*** (1.56, 6.45)	_ _ _	3.49*** (1.65, 7.40)	_ _ _
Nurse, nurse practitioner, physician assistant, or medic^b^	3.1 (2.3, 4.2)	10.4 (7.0, 15.2)	_ _ _	3.16*** (1.71, 5.84)	_ _ _	3.09*** (1.62, 5.88)	_ _ _
Social worker, counsellor, or psychotherapist	5.8 (4.7, 7.2)	8.4 (4.5, 15.0)	17.9 (11.9, 26.0)	1.66 (0.77, 3.56)	2.51** (1.35, 4.64)	1.57 (0.74, 3.35)	2.38** (1.29, 4.41)
Any professional help-seeking	22.5 (20.4, 24.8)	36.3 (27.0, 46.8)	66.3 (57.4, 74.2)	2.38*** (1.45, 3.91)	5.76*** (3.59, 9.23)	2.25*** (1.37, 3.69)	5.56*** (3.42, 9.03)

Notes. CI = confidence interval; AOR = adjusted odds ratio. AOR-1 models adjust for sex, race, marital status, total household income, education, and last military environment. AOR-2 models adjust for AOR-1 plus any child maltreatment and deployment-related traumatic event

^a^No cannabis use in the past 12-months was the reference category with an odds of 1.00

^b^To meet Statistics Canada data release guidelines, we were only able to examine seeking help from other medical doctors (e.g., cardiologist, gynecologist, urologist) or seeking help from a nurse, nurse practitioner, physician assistant, or medic in the past 12-months using a dichotomous version of the past 12-month cannabis use variable (i.e., no use/1 time use vs. used more than 1 time)

^*^p$$\le$$.05; **p$$\le$$.01; ***p$$\le$$.001

We also examined whether sex, any child maltreatment history, any DRTE, any past 12-month mental disorder, and any chronic pain condition moderated the relationship between past 12-month cannabis use and any perceived need for care or any past 12-month help-seeking behaviour. None of the interaction terms were statistically significant.

## Discussion

Past 12-month cannabis use among Veterans in the current study (16.7%) is similar to Canadian general population estimates before (15%) and after legalization changes (22%) (Health Canada, 2019). Importantly, 43.6% of Veterans who used cannabis reported daily use. Chronic, heavy use of cannabis is a risk factor for CUD and other harms (Hasin 2018; Hall 2014; Moore et al. 2007; Volkow et al. 2014; Windle et al. 2019). Cannabis using Veterans in Canada may represent a population in need of additional supports and services, given evidence of strong associations between cannabis use and current smoking, mental health disorders, and suicide behaviours, particularly Veterans who use cannabis on a regular basis (Turner and MacKillop 2021; Davis et al. 2018; Ecker et al. 2020; Hill et al. 2021; Sterniczuk and Whelan 2016).

Similar to previous studies, frequent past 12-month cannabis use was associated with PTSD, major depressive disorder, generalized anxiety disorder, social phobia, and panic disorder in the current study (Turner and MacKillop 2021; Ecker et al. 2020; Sterniczuk and Whelan 2016). In contrast, infrequent past 12-month cannabis use was not associated with most mental disorders (except PTSD) assessed in this study. Regular, but not infrequent, use of cannabis was also associated with current chronic pain conditions. Meta-analytic results also indicate an association between heavy cannabis use and suicidal behaviors (Borges et al. 2016). Canadian Veterans are disproportionately affected by chronic pain conditions in general (Thompson et al. 2016; VanTil et al. 2018; Perera et al. 2021), and among Veterans reimbursed for medical cannabis in particular (Reyes-Vélez et al. 2022). We were unable to differentiate medical from non-medical cannabis use in the current study, but many medical cannabis users also report recreational use (Browne et al. 2022; Carnide et al. 2021; Pacula et al. 2016). Providing health care providers with evidence-based information on the harms and benefits of using cannabis might help to facilitate transparent and non-judgmental conversations about cannabis use with their clients (Harris-Lane et al. 2025).

In addition to pain management, many Veterans report using cannabis to relieve PTSD and other mental health symptoms such as anxiety, depression, and stress (Reyes-Vélez et al. 2022; Sterniczuk and Whelan 2016; Windle et al. 2019). The cross-sectional design used for the current study limits causality discussions. Indeed, cannabis use might contribute to or exacerbate mental health symptoms, or cannabis use and other mental health problems might share a similar etiology (Hasin 2018). It could also be that cannabis use and mental health symptoms are completely unrelated, particularly among more infrequent users. A recent systematic review concluded that there may be more harms than benefits associated with cannabis use among Veterans, given the strong association of cannabis use with mental health disorders, other substance use, and suicidality (Turner and MacKillop 2021). However, other research suggests that Veterans report that cannabis use has beneficial impacts on their mental health (Sheehy et al. 2025), and has also been shown to reduce anxiety and depression symptoms in a small non-Veteran sample (Wolinsky et al. 2025). The dearth of evidence on the therapeutic benefits and harms of cannabis use among Veterans remains a serious limitation of the extant research (Turner and MacKillop 2021). Finding ways to reduce cannabis risk while evidence accumulates is important. Increasing awareness of lower risk cannabis use guidelines among Veterans and their health care providers is an important harm reduction approach to lowering risk (Harris-Lane et al. 2025).

In the current study, Veterans who used cannabis were more likely to perceive a need for care and to engage in past 12-month help-seeking than Veterans who did not use cannabis, with larger effect sizes noted for Veterans using cannabis on a more regular basis. Differences in perceived need for care were especially pronounced in the information, medication, counselling, and financial/housing assistance domains (i.e., more than 30% of infrequent users and 45% of regular users reported a perceived need in each of these domains). Facilitating access to supports and services across these domains may be an important prevention and intervention priority.

Veterans who used cannabis compared to those who did not were also more likely to seek help, and typically report to primary care providers for assistance (27.0% of infrequent users and 43.6% of regular users). After military service, Canadian Veterans transition to provincial healthcare coverage and out of the federally organized CAF health system. Importantly, only select mental health services are covered under provincial health insurance systems (St. Cyr et al. 2022), and less than 20% of Canadian Veterans receive services from Veterans Affairs Canada (VanTil et al. 2018). Representative data from the US indicates that most individuals with a past 12-month CUD do not receive any type of service for cannabis problems (i.e., only 7.2% received any type of service) (Hasin 2018). Therefore, primary care clinicians and other service providers should assess for cannabis use, including frequency, patterns, and motivations for use, among their Veteran clients. There is a high burden for Veterans from exposures to traumatic events (e.g., child abuse and DRTEs), and strong associations between traumatic events and cannabis use; (Afifi et al. 2023) accordingly, appropriate and timely referrals to supports and services are needed to better address the complex needs of Canadian Veterans.

The current study limitations also help inform future research directions. First, we could not differentiate between medical and non-medical cannabis use in the current study. Second, we lacked the statistical power to examine how more detailed information on the frequency of cannabis use related to major study variables, and lacked information regarding motives for use. We could also not assess whether respondents met criteria for CUD with the available data. Third, chronic pain and mental disorder assessments were based on self-reported data rather than clinician diagnoses, which may have biased the results. Fourth, the low prevalence of reported illicit drug use and non-medical use of prescription medications precluded the examination of cannabis use with illicit drugs in the current study. As well, we were only able to examine the association between any past 12-month alcohol use (yes or no) or any past 12-month binge drinking (yes or no) and cannabis use. More detailed information on alcohol use (e.g., frequency, quantity) and its association with cannabis use remains an important avenue for future research. Fifth, the items on perceived need for care and professional help-seeking were not specific to cannabis use (i.e., respondents were asked to report on whether they had sought help for problem with emotions, mental health, or the use of alcohol or drugs in the past 12-months). Sixth, the current results are based on a sample of Canadian Veterans, and may not generalize to CAF active duty military personnel who were not asked the cannabis use questions in the survey. Finally, the cross-sectional nature of the current study design makes inferences about causality impossible.

The current study highlights the strong association between regular cannabis use and mental health disorders, chronic pain, suicidality, and the use of nicotine among Canadian Veterans. The associations indicate Canadian Veterans who use cannabis on a regular basis may represent a population that might benefit from additional supports and services to better address their complex needs. Many conditions reported by Veterans in the current study are also the same conditions for which cannabis is medically authorized (Reyes-Vélez et al. 2022; Turner and MacKillop 2021); accordingly, more research on the therapeutic benefits and harms of cannabis use among Veterans appears critically needed (Turner and MacKillop 2021). Early identification and intervention with Canadian Veterans at risk of developing cannabis-related problems may help to improve their overall health and well-being.

## Data Availability

The dataset supporting the conclusions of this article can be accessed with permission from Statistics Canada. The data can be accessed through the Statistics Canada Research Data Centre. A formal process is in place for researchers to apply for access to the data only within a Statistics Canada Research Data Centre.

## Abbreviations

AOR: Adjusted odds ratio
CAF: Canadian Armed Forces
CAFVMHS: Canadian Armed Forces Members and Veterans Mental Health Follow-up Survey
CI: Confidence interval
CUD: Cannabis use disorder
DRTE: Deployment-related traumatic event
PNCQ: Perceived Need for Care Questionnaire
PTSD: Post-traumatic stress disorder
US: United States
